# Identification of genes encoding a novel ABC transporter in *Lactobacillus delbrueckii* for inulin polymers uptake

**DOI:** 10.1038/s41598-021-95356-1

**Published:** 2021-08-06

**Authors:** Yuji Tsujikawa, Shu Ishikawa, Iwao Sakane, Ken-ichi Yoshida, Ro Osawa

**Affiliations:** 1 Central Research Institute, ITO EN, Ltd., 21 Mekami, Makinohara, Shizuoka 421-0516 Japan; 2 Department of Bioresource Science, Graduate School of Agricultural Science, Kobe University, 1-1 Rokkodai, Nada, Kobe, 657-8501 Japan; 3 Department of Science, Technology and Innovation, Kobe University, 1-1 Rokkodai, Nada, Kobe 657-8501 Japan

**Keywords:** Bacteria, Bacterial evolution, Bacterial systems biology

## Abstract

*Lactobacillus delbrueckii* JCM 1002^T^ grows on highly polymerized inulin-type fructans as its sole carbon source. When it was grown on inulin, a > 10 kb long gene cluster *inuABCDEF* (Ldb1381-1386) encoding a plausible ABC transporter was suggested to be induced, since a transcriptome analysis revealed that the fourth gene *inuD* (Ldb1384) was up-regulated most prominently. Although *Bacillus subtilis* 168 is originally unable to utilize inulin, it became to grow on inulin upon heterologous expression of *inuABCDEF*. When freshly cultured cells of the recombinant *B. subtilis* were then densely suspended in buffer containing inulin polymers and incubated, inulin gradually disappeared from the buffer and accumulated in the cells without being degraded, whereas levan-type fructans did not disappear. The results imply that *inuABCDEF* might encode a novel ABC transporter in *L. delbrueckii* to “monopolize” inulin polymers selectively, thereby, providing a possible advantage in competition with other concomitant inulin-utilizing bacteria.

## Introduction

Inulin-type fructans (referred to, hereafter, as inulin) are common components of dietary fruits and vegetables, such as artichokes, chicory, bananas, garlic, and asparagus^1^. Inulin is a linear D-fructose polymer with β-(2–1) glycosidic bonds that is terminated by an α-(1–2)-glycosidic bond^2,3^-containing glucose molecule, in which the degrees of polymerization range from 2 to over 60^4^. Because of the β-(2–1)-linkages, inulin is not readily digested by humans but can be degraded and utilized by some intestinal bacteria with probiotic properties, such as lactobacilli and bifidobacterial. Inulin has thus been recognized as a prebiotic that can selectively stimulate probiotic bacterial growth, activity, or both, in the colon, thereby improving the host's health.

Probiotic bacteria are known to degrade inulin with extracellular enzymes, collectively termed inulinase or β-fructofuranosidase (SacA)^5^, into shorter fractions that include sucrose, fructose, and inulin with a degree of polymerization (DP) up to 6. These fractions are then taken up by the bacterial cells via membrane-bound proteins, known as sugar transporters, such as phosphotransferase system (PTS) and ABC transporter^6–12^. Tsujikawa et al.^13^ conducted an experiment in which *Lactobacillus paracasei* DSM 20,020 and *L. delbrueckii* JCM 1002^T^ were grown in the presence of inulin (DP of > 8) and found that the former degraded the inulin, releasing free fructose into the media during growth, which was clearly detectable by thin layer chromatography (TLC), whereas the latter did not show such extracellular fructose accumulation. The evidence suggested that JCM 1002^T^ did not degrade inulin extracellularly but transported it whole into its cells for further metabolism. The bacteria *Sphingomonas* sp. strain A1 has been reported to transport alginate, another highly polymerized carbohydrate, into their cells through the ABC transporter system^14^. Therefore, we were compelled to investigate if the *L. delbrueckii* is equipped with a similar macromolecule-transport system for inulin.

This study aimed to identify the genes responsible for the transport of highly polymerized inulin (DP of > 8), since this identification leads to the discovery of a novel sugar transporter. To this end, global gene transcription profiles were employed to nominate candidate genes involved in inulin transport. A wide region of DNA containing the transcriptional units of the most upregulated candidate genes in the presence of inulin was then introduced into the *amyE* region of *Bacillus subtilis* 168, which does not grow when inulin is the sole carbohydrate source. Any transformant that grew with inulin was densely suspended in PBS containing inulin and incubated. During the incubation, we monitored the disappearance of the inulin fractions from the PBS and their accumulation within the bacteria. Our results revealed the presence of the gene cluster that encodes a novel inulin transporter of JCM 1002^T^ required for the uptake of highly polymerized inulin-type fructans (DP of > 8).

## Results

### ***L. delbrueckii*** JCM 1002^T^ is capable of degrading and utilizing long-chain inulin

**Figure 1:**
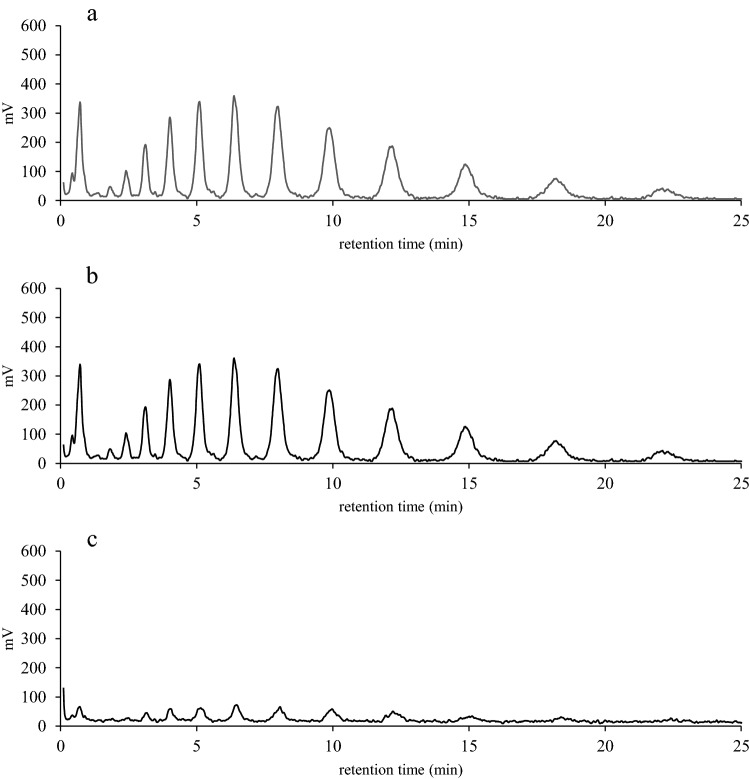
Comparison of extracellular Fuji FFSC inulin levels of JCM 1002^T^ cells. JCM 1002^T^ cells, which had been precultured in the presence of glucose or inulin, were incubated with Fuji FFSC inulin in PBS at 37 °C for 0 and 1 h, and the supernatants were analyzed by HPLC. Fuji FFSC inulin elution patterns for 0 (**a**) and 1 (**b**) h-incubation of the cells precultured in the presence of glucose, and 1 h-incubation of the cells precultured in the presence of inulin (**c**) are shown.

We first investigated how inulin was utilized by JCM 1002^T^ cells. The inulin used in the study consists of fructans of different degrees of polymerization (*i.e.* from 3 to 20) and presented a unique HPLC separation pattern of multiple peaks (Fig. 1a). When the cells were grown in the medium containing glucose were incubated with the inulin, there found no change in the separation pattern (Fig. 1b). In contrast, when the cells were grown in the medium containing the inulin, the separation pattern changed drastically with weakened signals of the peaks (Fig. 1c). The results suggested that that inulin could be degraded and utilized only by the cells grown with inulin.

### Selection of candidate genes involved in inulin transport by RNA-seq

JCM 1002^T^ was grown either in the presence of glucose or inulin for 6 h, and we performed the RNA-seq analysis to compare the transcriptome profiles. We selected genes belonging to the following two criteria stepwise; the first criterion consists of genes induced to more than double after 6 h of growth in the presence of inulin, and among them, the second criterion consists of genes repressed to less than half in the presence of glucose (Supplementary Fig. 1). We expected that this selection would allow us to narrow down genes induced by inulin specifically, but in fact, there were too many such genes for this to be possible. The difference in the transcriptome indicated that the gene sets involved in or affecting the metabolism of glucose and inulin are completely different genetic entities. We thus used cellobiose as an alternative carbon source, which is composed of 2 molecules of D-glucose linked by a b-1,4ʹ-glycosidic bond, and JCM 1002^T^ grew well in the presence of cellobiose as with inulin (Supplementary Fig. 2).

Subsequently, we compared the transcriptome profiles of JCM 1002^T^ cells grown either with cellobiose or inulin, indicating that the expression levels of the majority of genes did not change significantly (Supplementary Fig. 3). We analyzed a total of 1,587 genes to determine their FPKM value. As a result, only 51 genes satisfied the first criterion and the second criterion with cellobiose instead of glucose. We then screened the 51 genes with the third criterion where the genes tentatively encoding a transporter or whose function is unknown, and nominated a total of 22 candidate genes which might include genes responsible for the inulin transporting as listed in Supplementary Table 1. The increase in gene expression levels in inulin compared to cellobiose was between 2.1-fold and 3.5-fold.

To validate the expression data obtained from the RNA-seq experiments, the transcript levels of the 22 candidate genes were measured by RT-PCR (Table 1). The expression of 16 genes was reproducibly induced in the presence of inulin compared with cellobiose (between 1.0-fold and 4.4-fold). Among them, Ldb0442 (3.56-fold) and Ldb1384 (4.44-fold) showed the highest expression increase and were the most interesting candidate genes.

**Table 1:** Transcription levels of 22 candidate genes on inulin compared to cellobiose by RT-PCR.

Locus_Tag	Product	Fold change^(a)^	
		Inuin 0 → 6 h	Inulin/celobiose
Ldb1384	ABC transporter permease	1.87 ± 0.22	4.44 ± 0.34
Ldb0442	PTS sugar transporter subunit IIC	1.98 ± 0.12	3.56 ± 0.31
Ldb1299	ABC transporter permease	1.82 ± 0.25	2.34 ± 0.28
Ldb1298	Amino acid ABC transporter ATP-binding protein	1.76 ± 0.11	2.87 ± 0.19
Ldb0035	Putrescine/spermidine ABC transporter ATP-binding protein	1.11 ± 0.04	2.26 ± 0.27
Ldb1652	Hypothetical protein	1.44 ± 0.19	1.92 ± 0.14
Ldb0276	Peptide ABC transporter substrate-binding protein	1.44 ± 0.11	1.92 ± 0.18
Ldb1221	M23 family peptidase	1.54 ± 0.21	1.88 ± 0.20
Ldb0331	Metal ABC transporter ATP-binding protein	1.89 ± 0.23	1.57 ± 0.13
Ldb0021	ABC transporter ATP-binding protein	0.98 ± 0.12	1.43 ± 0.08
Ldb0154	ABC transporter ATP-binding protein	1.25 ± 0.08	1.38 ± 0.09
Ldb1651	Hypothetical protein	1.12 ± 0.09	1.34 ± 0.12
Ldb1993	Hypothetical protein	0.89 ± 0.04	1.33 ± 0.07
Ldb0476	Hypothetical protein	2.1 ± 0.31	1.23 ± 0.08
Ldb1385	ABC transporter ATP-binding protein	1.01 ± 0.12	1.12 ± 0.14
Ldb1386	ABC transporter ATP-binding protein	1.23 ± 0.13	1.01 ± 0.07
Ldb0330	ABC transporter permease	0.76 ± 0.05	0.95 ± 0.06
Ldb0295	Purine permease	0.99 ± 0.09	0.86 ± 0.09
Ldb1990	Hypothetical protein	1.13 ± 0.28	0.67 ± 0.04
Ldb0973	ABC transporter ATP-binding protein	0.99 ± 0.03	0.67 ± 0.07
Ldb0974	ABC transporter ATP-binding protein	0.81 ± 0.11	0.66 ± 0.08
Ldb1574	MFS transporter	0.22 ± 0.02	0.34 ± 0.04

^(a)^Inulin 0 → 6 h; the range of the gene expression levels on inulin from 0 to 6 h, Inulin/Cellobiose; the range of the gene expression level on inulin compared to cellobiose.

### Identification of genes involved in inulin transport

**Figure 2:**
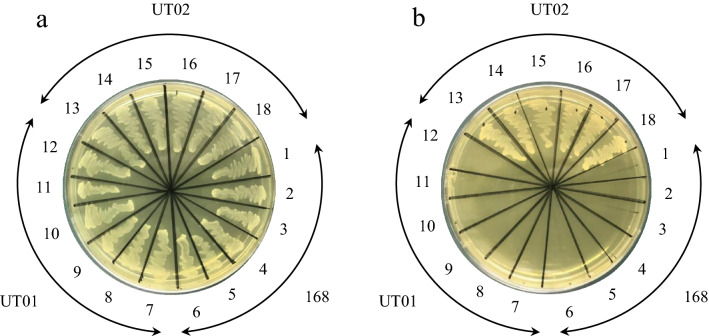
Introduction of the Ldb1381-1386 region from JCM 1002^T^ allowed *B. subtilis* to utilize inulin. Six clones of UT001 (*amyE*::Ldb0438-0442, No. 7-12) and UT002 (*amyE*::Ldb1381-1386, No. 13-18) obtained just after transformation were grown with the parental 168 strain (No. 1-6) on minimal medium containing (**a**) glucose or (**b**) inulin at 37 °C for 48 h.

To examine whether the two candidate genes are responsible for the inulin transporter, *Bacillus subtilis* 168, which, like *L. delbrueckii*, belongs to Firmicutes and is easy to genetically manipulate, was utilized. Conveniently for this validation, *B. subtilis* 168 is incapable of growing in the presence of inulin (Fig. 2b 1–6). To investigate the functions of the candidate genes, wide regions containing the transcriptional units for Ldb0442 (Ldb0438-0448) and Ldb1384 (Ldb1381-1386), including adjacent intergenic regions, were introduced into the *amyE* region of *B. subtilis* 168 (designated UT01 and UT02 strains, respectively) to test if the exogenously introduced gene clusters would allow *B. subtilis* 168 to utilize inulin (Fig. 2).

Six clones of UT01 (*amyE*::Ldb0438-0448) and UT02 (*amyE*::Ldb1381-1386), together with the 168 strain, were cultured on glucose or inulin as a sole carbon source. All clones grew well in the presence of glucose as expected (Fig. 2a). Although no growth was observed for any clone of the parental 168 strain or UT01, all clones of UT02 grew well in the presence of inulin, indicating that the Ldb1381-1386 region is responsible for inulin unitization, probably involving inulin transport, and thus the region was designated *inuABCDEF* (Fig. 2b).

To confirm that the *inuABCDEF* introduced with a chloramphenicol cassette (*cat*), but no other region(s), in the UT02 strain was responsible for the inulin-utilization phenotype, a back-cross experiment was performed by introducing the *inuABCDEF* with the *cat,* which was amplified from the UT02 strain (Supplementary Fig. 4). As a result of comparing colony formation on minimal medium agar plates containing chloramphenicol, inulin, or both, almost the same number of transformants appeared under all conditions, proving that those that can use inulin always acquire chloramphenicol resistance as well, and that inulin can be used as long as *inuABCDEF* is acquired.

### Inulin is transported by an ABC transporter encoded by *inuABCDEF* in *B. subtilis* cells

**Figure 3:**
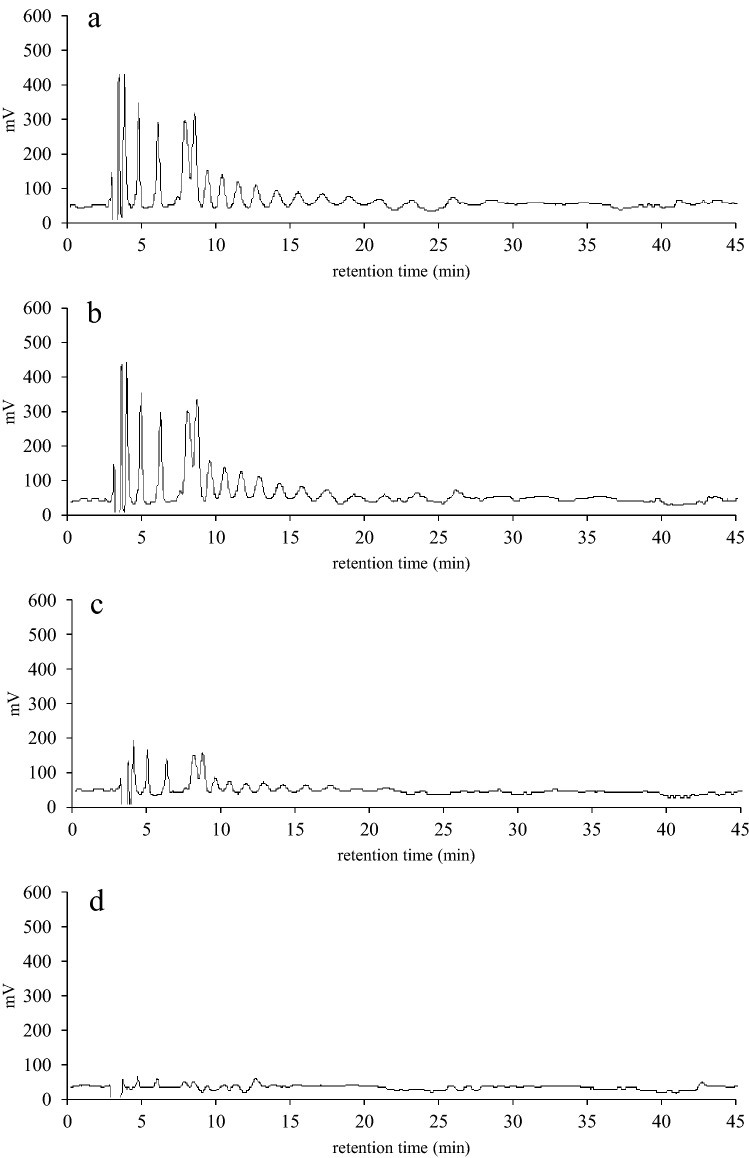
Comparison of extracellular inulin of *B. subtilis* 168 and UT02 (Ldb1381-1386-introduced 168 strain) cells. The cells were incubated with inulin in PBS at 37 °C for 0.5, 1, and 2 h, and the supernatants were analyzed by HPLC. Inulin elution patterns for 2 h- incubation of *B. subtilis* 168 cells (**a**), and 0.5 (**b**), 1 (**c**), and 2 (**d**) h-incubation of UT02 cells are shown.

**Figure 4:**
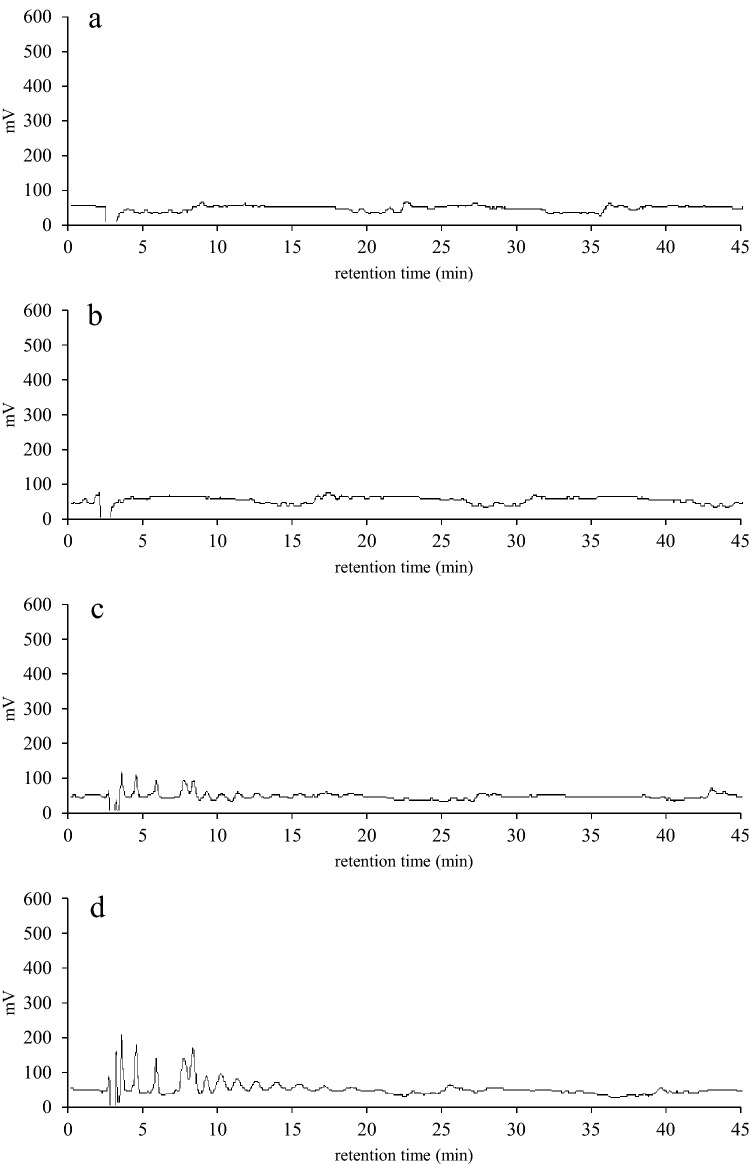
Comparison of intracellular inulin of *B. subtilis* 168 and UT02 (Ldb1381-1386-introduced 168 strain) cells. The cells prepared as described in Fig. 3 were lysed and disrupted by sonication, and intracellular inulin polymers in the supernatants were analyzed by HPLC. Inulin elution patterns for 2 h-incubation of *B. subtilis* 168 cells (**a**) and 0.5 (**b**), 1 (c), and 2 (d) h-incubation of UT02 cells are shown.

To investigate inulin transport into the UT02 cells, *B subtilis* cells with (UT02) or without (168 strain) an ABC transporter homolog encoded by *inuABCDEF* were incubated in PBS with inulin, and the extracellular and intracellular inulin levels were analyzed using HPLC. In the case of the extracellular inulin of 168-strain cells, the HPLC pattern of initially observed multiple peaks corresponding to inulin polymers did not change, even 2 h after the start of the incubation; whereas, for the UT02 cells, the peaks began to shrink after 0.5 h and disappeared after 2 h (Fig. 3). In contrast, in the case of the intracellular inulin of 168-strain cells, no peak corresponding to inulin emerged, even after 2 h, while for the UT02 cells, inulin peaks were clearly present after 1 h and increased after 2 h (Fig. 4). These results indicated that *inuABCDEF* encoding the ABC transport homolog in the UT02 stain was responsible for inulin transport.

### Substrate specificity of the ABC transporter encoded by *inuABCDEF*

**Figure 5:**
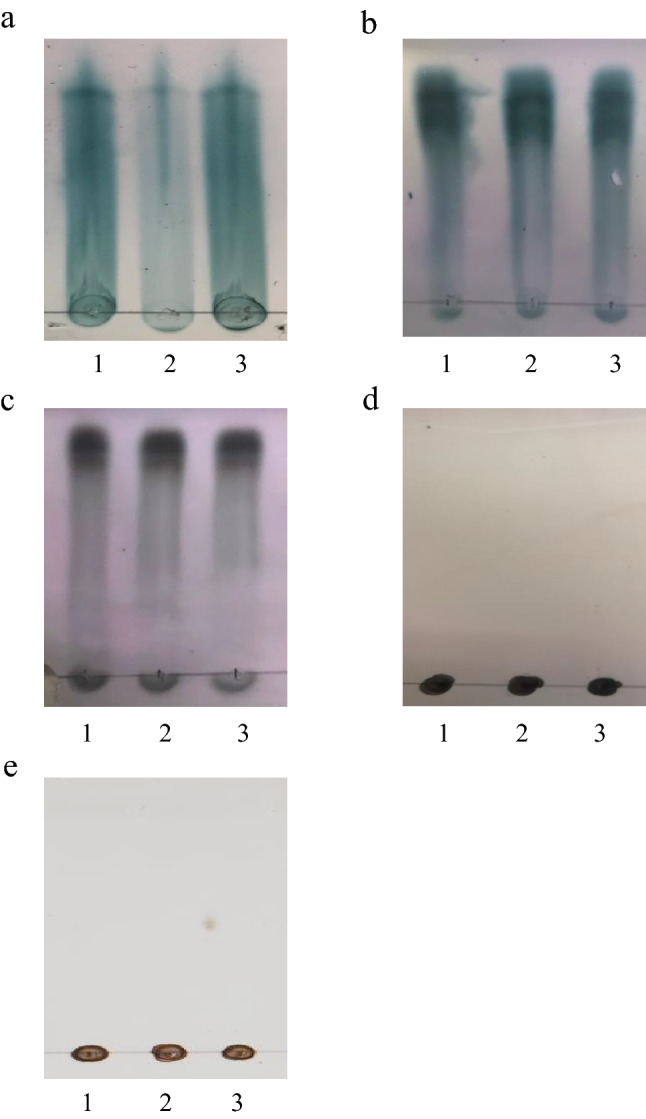
Extracellular amounts of oligosaccharides or polysaccharides measured by TLC. Sugar compositions of the supernatants were determined after cells of *B. subtilis* 168 or UT02 were densely suspended in PBS containing inulin (**a**), IMO (**b**), XOS (**c**), alginate (**d**) or levan (**e**) and incubated at 37 °C for 2 h. Lane 1, *B. subtilis* 168 (after 2 h); lane 2, UT02 (after 2 h); lane 3, Control (1%, wt/vol).

To investigate the substrate specificity of the transporter encoded by *inuABCDEF*, the ability of the UT02 strain cells to take up other oligosaccharides (isomalto-oligosaccharide: IMO and xylo-oligosaccharide: XOS) and polysaccharides (alginate and levan) was examined by analyzing extracellular levels using TLC. As predicted, the amount of extracellular inulin remained unchanged after *B. subtilis* 168 cells were incubated for 2 h (Fig. 5a lane 1), while that decreased after UT02 cells were incubated for 2 h (Fig. 5a lane 2), On the other hand, there was no change to the amount of extracellular IMO after UT02 cells or the *B. subtilis* 168 cells were incubated for 2 h (Fig. 5b). The same applied to the XOS (Fig. 5c), alginate (Fig. 5d), and levan (Fig. 5e). These results indicated that *inuABCDEF* is not involved in the transport of IMO, XOS, alginate, or levan but is specifically associated with inulin transport.

### Introduction of the *inuABCDEF* into 168 mutant strains

A homology search showed that *inuABCDEF* only encodes an ABC transporter and not any genes involved in inulin degradation, even though the *inuABCDEF*-introduced *B. subtilis* strain, UT02, grew in the presence of inulin. This fact implies that the intracellular inulin transported by Ldb1381-1386 was utilized after degradation by an enzyme that *B. subtilis* 168 originally possessed.

**Figure 6:**
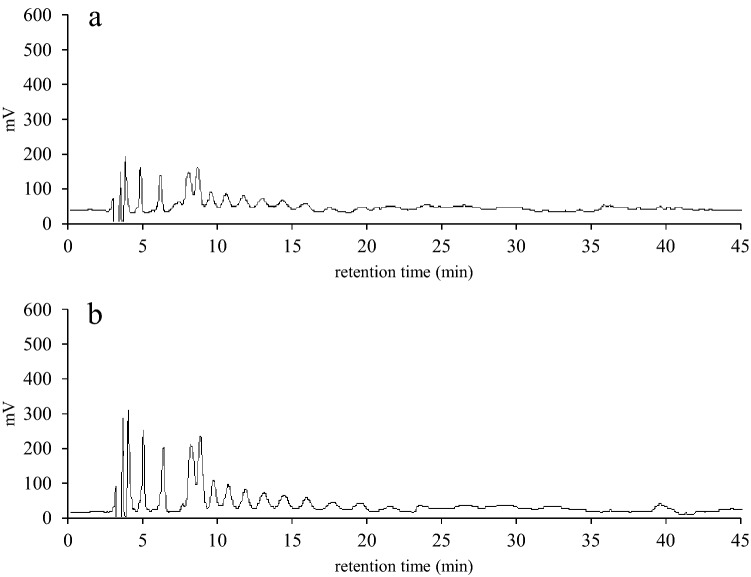
Comparison of intracellular inulin of UT02 (Ldb1381-1386 introduced 168 strain) and UT05 (Ldb1381-1386 introduced 168 strain with *sacC* deletion) cells. The cell lysates were prepared as described in Fig. 4, and intracellular inulin polymers were analyzed by HPLC. Inulin elution patterns after 2 h -incubation of UT02 (**a**) and UT05 (**b**) are shown.

SacC has been reported to be a levanase that is not only capable of hydrolyzing levan but also inulin and sucrose^15^. According to UniProt, SacA and LevB belong to the same glycosyl hydrolase 32 family as SacC. In the case of LevB, endo-activity specifically hydrolyzing (β-2,6) fructosyl bonds has been reported^16^. Thus, to examine if these enzymes were responsible for the inulin degradation in the UT02 strain, *sacA*, *levB,* and *sacC* genes were deleted in the UT02 strain, and the resultant clones were designated UT03, UT04, and UT05, respectively (Supplementary Fig. 5). UT03 (*△sacA*) and UT04 (*△levB*) were seen to grow in the presence of inulin, while UT05 (*△sacC*) showed no growth (Supplementary Fig. 6). Consistent with this phenotype, amount of the intracellular inulin polymers in the UT05 cells were increased compared to that in the UT02, indicating that the intracellular inulin in the UT05 cells was not digested in the absence of SacC as done in the UT02 cells (Fig. 6).

## Discussion

JCM 1002^T^ grew well in the presence of glucose, cellobiose or inulin. According to the GenBank, JCM 1002^T^ possess glucose PTS (accession no. CAI98592) and cellobiose PTS (accession no. CAI97373 and CAI98861). Therefore, it is thought that JCM 1002^T^ take up glucose and cellobiose into cells via PTS. In this study, it is possible to nominate candidate genes involved in a novel inulin transporter by differential gene transcription^10,11^ between glucose-grown cells and inulin-grown cells. However, expression levels of enormous genes showed a significant difference, and we failed to narrow down the candidate genes induced by inulin specifically. This failure was probably due to carbon catabolite repression (CCR)^17^. CCR is one of the most fundamental and highly conserved mechanisms in bacteria to repress various genes in the presence of a preferred carbon source such as glucose^18^. JCM 1002^T^ belongs to low-GC Gram-positive bacteria. Thus, its CCR may depend on catabolite control protein A (CcpA) as the principal DNA-binding regulator. CcpA forms a complex with P-ser-HPr to bind to its target sites with the consensus sequence named *cre* to repress the genes under CCR^19^. We found that most of the genes repressed in the presence of glucose were associated *cre* sequences. However, for a proper discussion of CCR, the results obtained in this study are still preliminary, and the further discussion of CCR remains for another time in the future.

As a result of RT-PCR, Ldb0442 and Ldb1384 were selected as the leading candidate form the 22 candidate genes. And then, by use of a complementation test using *B. subtilis*, which is originally not able to utilize inulin, as a host, a gene cluster containing the Ldb1384 was finally found to be a candidate for an inulin transporter, since the cluster-introduced strain became to utilize an inulin as a sole carbon source. Although for some *Lactobacillus*, genetics to identify genes involved in utilization of inulin-type fructans from candidate genes exist, such as plasmid integration via homologous recombination^10^ and Cre-lox-based mutagenesis system^11^, however, such methods could not be applied to the JCM 1002^T^ so far. Thus, this system to use the *B. subtilis* as a host to test function of genes would be useful not only for JCM 1002^T^ but also for some bacteria whose genetics has not been established.

In this study, we demonstrated that the gene cluster Ldb1381-1386, designated *inuABCDEF*, encodes a novel inulin transporter of JCM 1002^T^ required for the uptake of highly polymerized inulin-type fructans (DP of > 8). There are some reports of bacterial strains that can directly transport short inulin-type fructans into their cells. For example, *L. paracasei* 1195^9^ and *L. acidophilus* NCFM^10^ transport fructo oligo saccharides (FOS) with degrees of polymerization (DP) from 3 to 5 into their cells via the ABC transporter and intracellularly degrade them. *L. plantarum* WCFS1 can take up 1-kestose (DP 3) and nystose (DP 4) through a sucrose-transport system^20^. Some *Bifidobacterium* strains can transport oligofructose (DP 2 to 8) directly into their cells^21–23^. However, with regards to transporters that directly take up highly polymerized inulin-type fructans (DP of > 8), Widodo et al.^24,25^ reported that *Lactobacillus casei* AP takes up inulin via the PTS or ABC transporter, degrading it intracellularly. However, the inulin-transport-associated genes have not been identified. Therefore, as far as we know, this is the first report on the identification of a gene cluster responsible for the transport of highly polymerized inulin-type fructans (DP of > 8) into bacterial cells.

According to GenBank, *inuAB* (Ldb1381-1382), *inuCD* (Ldb1383-1384), and *inuEF* (Ldb1385-1386) are annotated to encode oligopeptide ABC transporter substrate binding protein (accession no. CAI98182 and CAI98183), oligopeptide ABC transporter permease (accession no. CAI98184 and CAI98185), and oligopeptide ABC transporter ATP-binding protein (accession no. CAI98186 and CAI98187), respectively. Therefore, *inuABCDEF* may also have “oligopeptide ACB transporter” function in addition to inulin transporter function. Reportedly, putative oligopeptide ATP transporters are responsible for sugar and oligosaccharide transport in *Thermotoga maritima*^26^. Although, as far as we know, there is no evidence that the oligopeptide ABC transporter functions as a polysaccharide transporter. To investigate the substrate specificity of the transporter encoded by *inuABCDEF*, we introduced *inuABCDEF* into the 168 strain, and the bacteria’s ability to take up oligosaccharides (IMO and XOS) and alginate were investigated by TLC. The results suggested that *inuABCDEF* does not encode the transporter for these sugars. IMO is a mixture of glucose oligomers with α-(1–6)-linkages, while XOS is a mixture of xylose with β-(1–4)-linkages. Alginate is composed of blocks of mannuronic acid residues, blocks of guluronic acid residues, and blocks of alternating M and G residues. Moreover, our results show that levan does not seem to be recognized by *inuABCDEF*. Inulin is a D-fructose polymer linked by β-(2–1)-glycosidic bonds with a terminal glucose moiety that is linked by an α-(1–2)-glycosidic bond, as in sucrose. Correspondingly, levan possesses β-(2–6)-linkages as its skeleton, with branching β-(2–1)-linkages^27^. Therefore, the ABC transporter encoded by *inuABCDEF* may recognize β-(2,1)-fructosyl-linkages or 1-alpha-D-glucopyranosyl-2-beta-D-fructofuranoside. A future investigation of the specific substrate of the transporter will be necessary to elucidate the function of this novel transporter in more detail.

This study showed that JCM 1002^T^ has a dedicated transportation system for taking up whole inulin polymers. Moreover, as shown in our last report, JCM 1002^T^ grows better on inulin-type fructans than on fructose^13^. There are some cases in which bacteria grow better when fed oligosaccharides than their degradation products. For example, *Bifidobacterium animalis* DN-173 010 grows on inulin-type fructans Raftilose Synergy1 and Raftilose P95 (powders containing inulin and enzymatically digested inulin, respectively) but not on fructose. This suggests the adaptation of the strain to oligosaccharide metabolism provides advantages when competing with other microorganisms in the human gut, where oligosaccharides are the main energy resource^28^. *L. delbrueckii* is derived from plants, and inulin exists in a variety of plants, such as some fruit, artichokes, chicory, garlic, and asparagus in natura^29^. Plants are internally inhabited by diverse microbial communities, including bacterial, archaeal, fungal, and protistic taxa^30^, which represent the many organisms that *L. delbrueckii* needs to compete with for survival in the ecosystem. For this reason, in plants where inulin is present, *L. delbrueckii* may have evolved the ability to transport whole inulin polymers directly into their cells to out-compete other concomitant inulin-utilizing bacteria, as the extracellular hydrolysis of inulin provides monosaccharides for opportunistic competitors. As far as we have been able to determine, there is no gene cluster in any other species that could be considered identical to *inuABCDEF* based on sequence homology. In addition to *Bifidobacterium animalis* DN-173 010, other bacteria that demonstrate unique sugar transportation according to each environment have been frequently reported. For example, human milk oligosaccharide-specific ABC transporters of *Bifidobacterium* help in the adaptation and dominance of the bacteria in the infant gut ecosystem^31^. *L. plantarum* isolated from humans*,* unlike the strains isolated from fermented food, lack the genes for FOS metabolism, indicative of their evolution and adaptation to different environmental niches^32^. In their probiotic roles, *L. delbrueckii* are reportedly responsible for enhanced host immune responses and have antioxidative activity^33–35^; therefore, *L. delbrueckii* promotes GI health in humans and other animals. However, some authors have reported that *L. delbrueckii* is rarely found among the gut microbiota after ingestion because of its limited capability to survive the restrictive conditions of GI digestion^36,37^. Other researchers have provided evidence that some *L. delbrueckii* strains can survive GI transit, but the number of bacteria decreases considerably in those cases^38^. If any of the probiotic strains of *L. delbrueckii* have a dedicated transportation system for the more highly polymerized inulin-type fructans, it may be possible to use such sugars as prebiotic supplements to selectively stimulate their growth in the host’s digestive tract, especially as host cells have difficulty absorbing and utilizing these fructans.

In conclusion, by selecting 22 candidate genes with RNA-seq and RT-PCR and identifying those involved in inulin utilization by introducing them into the *amyE* region of *B. subtilis* 168, we showed that *inuABCDEF* encodes a novel inulin transporter that directly transports inulin into the JCM 1002^T^ cells. This novel inulin transporter may form part of the system through which *L. delbrueckii* gains an ecological advantage in bacterial communities: those bacteria only degrading inulin extracellularly may, although temporally, release sizeable amounts of fructose molecules, which are then readily “scavenged” by other bacteria that only utilize fructose; whereas strains, like *L. delbrueckii*, that take up whole inulin for intracellular digestion can “monopolize” inulin resources.

## Methods

### Bacterial strains, media, and substrates

The bacterial strains used in this study were *L. delbrueckii* JCM 1002^T^*, Bacillus subtilis* YK05, and *B. subtilis* 168. JCM 1002^T^ was stored at − 80 °C in de Man-Rogosa-Sharpe (MRS) broth (Oxoid, Basingstoke, United Kingdom), and *Bacillus* strains were stored at − 80 °C in LB broth (Oxoid, Basingstoke, United Kingdom) until use. mMRS^39^ was used as the basal fermentation medium throughout this study. The pH of the medium was adjusted to 6.5 before sterilization (121 °C for 15 min). Glucose, cellobiose, or inulin (Wako Pure Chemical Industries) was added to mMRS as a carbon source. The DP of inulin (Wako Pure Chemical Industries) is reported to vary between 3 and 60 according to information supplied by the company. In all cases, these sugars were sterilized by membrane filtration using Millex Syringe Filter Units (pore size, 0.45 µm) and added aseptically to the sterile mMRS medium. Minimal medium was used to investigate the utilization of sugar by transformants. Minimal medium consisted of 1% glucose, 0.5% Spizizen’s minimal salts^40^, 1% tryptophan (5 mg/ml), 0.1% trace element solution, 0.002% FeCl_3_·4H_2_O, 0.002% MnSO_4_·5H_2_O, and 1.5% agar. Each liter of trace element solution contained 0.73 g CaCl_2_·2H_2_O, 0.36 g ZnS_4_·7H_2_O, CuSO_4_·5H_2_O 0.065 g, 0.06 g CoCl_2_·6H_2_O, and 0.06 g Na_2_MoO_4_·2H_2_O. Spizizen’s minimal salts were sterilized at 121 °C for 20 min, and others were sterilized by membrane filtration, then they were added to the sterilized medium to the desired concentration.

### Fermentation experiments

JCM 1002^T^ was cultured anaerobically in MRS broth at 37 °C for 12 h, after which the cultures were centrifuged at 15,000 rpm for 5 min. The bacterial pellet was then washed once with phosphate-buffered saline (PBS, 0.8% NaCl, 0.02% KH_2_PO_4_, 0.115% Na_2_HPO_4_ pH 7.4) and re-suspended in PBS until the optical density at 660 nm (OD660) reached 0.4, followed by spotting 50 µL of this suspension onto 5 mL of mMRS containing 2% glucose (wt/vol), 2% cellobiose (wt/vol), or 2% inulin (wt/vol). Incubations of the media were performed anaerobically at 37 °C for up to 48 h, during which their OD660 were measured at 3, 6, 9, 12, 24, and 48 h after incubation. Anaerobic conditions were provided using AnaeroPack-Anaero (Mitsubishi Gas Chemical Co., Inc., Tokyo, Japan).

### RNA-seq

JCM 1002^T^ cells were cultured in 5 mL of mMRS containing 1% glucose (wt/vol), 1% cellobiose (wt/vol) or 1% inulin (wt/vol) anaerobically at 37 °C for 6 h. After centrifugation at 15,000 rpm for 5 min, the bacterial cells were harvested, and total RNA was extracted and purified using the Bacterial RNA kit (OMEGA) according to the protocol provided by the manufacturer. Quality control of each RNA sample was performed with an Agilent 2100 Bioanalyzer (Agilent Technologies, USA). The cDNA libraries were constructed using NEBNextUltra RNA library Prep Kit (NEB, USA) and submitted for sequencing using Illumina Hiseq 4000 (Illumina). Library construction and sequencing were performed by Allwegene Biotechnology Co., Ltd. (Beijing, China). FPKM values for each gene and differentially expressed genes were analyzed with Cufflinks v2.2.131. The differentially expressed genes between glucose and inulin and between cellobiose and inulin were identified considering both fold changes, respectively.

In RNA-seq expression data, the genes that satisfied the following three criteria were selected as candidate genes for an inulin transporter.

1. Genes whose transcription level was at least twofold after 6 h of incubation with inulin.
2. Genes whose transcription level was at least twofold higher after 6 h of growth with inulin than either with glucose or cellobiose.
3. Genes predicted to encode a transporter, as judged by homology, or genes whose function is unknown according to the GenBank.

### Real-time RT-PCR

JCM 1002^T^ cells were inoculated into 5 mL of mMRS containing 1% cellobiose (wt/vol) or 1% inulin (wt/vol) and cultured anaerobically at 37 °C for 6 h. After centrifugation at 15,000 rpm for 5 min, the bacterial cells were harvested and homogenized in ISOGEN (Nippon Gene, Tokyo, Japan), and total RNA was extracted according to the manufacturer's instructions. cDNA was synthesized using a PrimeScript II Reverse Transcriptase according to the manufacturer’s instructions. The 20-µl reaction solution consisted of 2 µl of template, 10 µl of SYBR Premix Ex Taq, 0.4 µl of each primer (10 µM), and 0.4 µl of ROX Reference Dye. PCR amplification was performed as follows: predenaturation for 1 cycle at 95 °C for 30 s, 40 cycles at 95 °C for 5 s, and 60 °C for 30 s using a Thermal Cycler Dice Real Time System Lite (Takara, Shiga, Japan). Cycle threshold values for candidate genes were normalized to those of housekeeping genes Ldb0394 (*fusA*). Relative expression levels were calculated using the 2^−ΔΔCt^ equation^41^. Primers used to validate the expression data obtained from the RNA-seq experiment are shown in Supplementary Table 2.

### Thin layer chromatography (TLC)

To determine the extracellular amounts of IMO, XOS, alginate, or levan, we used TLC (Merck, silica gel 60 plate). Briefly, *B. subtilis* 168 or UT02 strains were cultured at 37 °C for 24 h in 50 ml of mMRS containing 1% glucose (wt/vol) or 1% inulin (wt/vol), respectively. The cells were collected, washed three times, suspended in 10 ml of PBS containing 1% IMO (wt/vol), XOS (wt/vol), alginate (wt/vol), levan (wt/vol), or inulin (wt/vol), and incubated at 37 °C for 2 h. After centrifugation at 15,000 rpm for 5 min, the supernatant was spotted, along with inulin standard (1%, wt/vol), onto different lanes of a TLC plate. The plates were developed in a 1-butanol/2-propanol/ethanol/water (3:2:3:4) solvent. Spots were visualized by spraying the plates with p-anisaldehyde (contains acetic acid, H_2_SO_4_) ethanol solution (Tokyo Chemical Industry, Tokyo, Japan) and heating them at 160 °C for several minutes.

### Extracellular and intracellular inulin analysis by High-Performance Liquid Chromatography (HPLC)

To investigate extracellular Fuji *Fusarium fujikuroi* species complex (FFSC) inulin levels for the JCM 1002^T^ cell culture, the strain was cultured 50 ml of in mMRS containing 1% glucose (wt/vol) or 1% inulin (wt/vol) at 37 °C in for 24 h. Bacterial cells were collected, washed three times, and suspended in 10 ml of PBS containing 1% Fuji FFSC inulin (Fuji Nihhon Seito Co., Japan) as the inulin. After incubation at 37 °C for 1 h, the supernatant was recovered by centrifugation at 15,000 rpm for 5 min and then employed in the qualitative analysis of Fuji FFSC inulin by HPLC (Waters Acquity UPLC H-Class) equipped with refractive index (RI) detector (Waters 2414) and Cosmosil PBr column [10 mm (inner diameter or ID) × 250 mm] (Nacalai Tesque). The temperature of column and RI detector was set to 30 and 40 °C, respectively. The mobile phase was metanol/water composition of 15/85 (v/v) at a flow rate of 0.9 ml/min. 10 µl of the sample was filtered with a 0.45 µm syringe filter (SupraPure; Recenttec, Tokyo, Japan) and injected to the column. The Fuji FFSC inulin, a commercial powder made with sugar as the raw material, contains inulin (86.8%, wt/wt), another glucide (6.6%, wt/wt), and water (6.6%, vol/wt). The average degree of polymerization (DP) of the Fuji FFSC inulin chains is reported to vary between 3 and 20, with an average of 8.

To investigate levels of extracellular inulin for *B. subtilis* cells, 168 and UT02 strains were cultured at 37 °C for 24 h in 50 ml of mMRS containing 1% glucose (wt/vol) and 1% inulin (wt/vol), respectively. The cells were collected, washed three times, suspended in 10 ml of PBS containing 1% inulin (wt/vol) and incubated at 37 °C for 0.5, 1, and 2 h. The supernatants were analyzed by HPLC as described above.

To investigate the intracellular inulin of *B. subtilis* 168 cells, 168 strain was cultured at 37 °C for 24 h in 50 ml of mMRS containing 1% glucose (wt/vol), while UT02 and UT05 strains were cultured at 37 °C for 24 h in 50 ml of mMRS containing 1% inulin (wt/vol). The cells were prepared as described above, lysed in 0.5 ml buffer (50 mM EDTA pH 8.5, 0.05% N-laryl sarcosine) containing 2 mg/ml of lysozyme (Sigma) and 3 U/ml mutanolysin (Sigma) at 37 °C for 16 h, and disrupted by sonication with glass beads for 30 s (MSE 150 W ultrasonic disintegrator, MSE, Crawley, Sussex, UK). Then, the inulin in the supernatants, which was separated by centrifugation (at 15,000 rpm for 5 min), was analyzed by HPLC as described above.

### Introduction of candidate genes into *B. subtilis*

Ldb1381-Ldb1386 or Ldb0438-Ldb0448 was introduced into the *amyE* region of *B. subtilis* 168 by a double-crossover event and designated UT01 and UT02, respectively (Supplementary Fig. 7). The 5’ and 3’ ends of the *amyE* gene were amplified from *B. subtilis* 168 by primer sets for cat-amyEF-f1/-r, and the chloramphenicol resistance gene (*cat*) cassette was amplified from *B. subtilis* YK05 by a primer set for cat-amyEB-f/-r1. Ldb0438-0448 and Ldb1381-1386, including adjacent intergenic regions, were amplified from the JCM 1002^T^ genome by primer sets for Ldb1380F/Ldb1387R and Ldb0437/Ldb0448R, respectively. Four PCR products of the 5′end of the *amyE*, Ldb0442, or Ldb1384 region, *cat* cassette, and the 3’ end of *amyE* were then assembled using the Gibson Assembly Master Mix (New England Biolabs E2611S) according to the manufacturer's instructions. The primers used are listed in Supplementary Table 3.

The assembled PCR fragments were then introduced into the *amyE* region by transformation^42^, and transformants were selected on minimal medium containing 1% glucose (wt/vol) and chloramphenicol (5 µg/ml) at 37 °C for 48 h. Six clones were then transferred to minimal agar media containing 1% glucose (wt/vol) or 1% inulin (wt/vol) and incubated at 37 °C for 48 h.

### Back-cross experiment

The Ldb1381-Ldb1386 region of the UT02 strain was amplified by a primer set for Ldb1380F/Ldb1387R, and the PCR product was introduced into *B. subtilis* 168 as described above. Transformants were selected on minimal medium containing chloramphenicol (5 µg/ml), 1% inulin (wt/vol), or both, at 37 °C for 48 h.

### Identification of genes involved in inulin degradation in *B. subtilis*

Deletion mutants of *sacC*, *levB,* and *sacA* were obtained by substitution with the erythromycin resistance cassette from the BKE^43^ collection in the National BioResource Project. The deletions were introduced into the UT02 strain and selected on minimal medium containing erythromycin (1 µg/ml) and lincomycin (12.5 µg/ml) at 37 °C for 48 h. Then, transformants were inoculated into minimal medium containing 1% glucose (wt/vol) or 1% inulin (wt/vol), and growth was observed after 48 h incubation at 37 °C.

## Supplementary Information

Supplementary Information 1.

Supplementary Information 2.

## Data Availability

The datasets used and analyzed during the current study are available from the corresponding author on reasonable request.
