# Supplementary material for: Identification of genes encoding a novel ABC transporter in Lactobacillus delbrueckii for inulin polymers uptake
Source: Sci Rep. 2021 Aug 6;11:16007. doi: 10.1038/s41598-021-95356-1 (PMC8346543; doi:10.1038/s41598-021-95356-1)
Supplement: Supplementary file 1 — Supplementary Information 1. [file 41598_2021_95356_MOESM1_ESM.docx]

**Identification of genes encoding a novel ABC transporter in *Lactobacillus delbrueckii* for inulin polymers uptake**

Yuji Tsujikawa, Shu Ishikawa, Iwao Sakane, Ken-ichi Yoshida, and Ro Osawa

Supplementary Figure

**Supplementary Fig. 1. Scatter plot of the RNA-seq expression data between glucose and inulin.**

**Supplementary Fig. 2. Growth curves of *L. delbrueckii* JCM 1002^T^ on mMRS containing glucose, cellobiose, or inulin.** OD = optical density.

**Supplementary Fig. 3. Scatter plot of the RNA-seq expression data between cellobiose and inulin.**


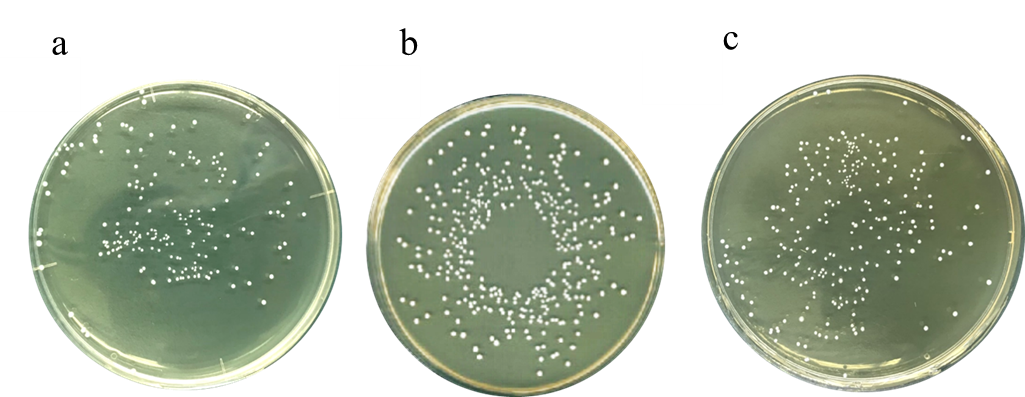


**Supplementary Fig. 4. Back-cross experiment.** Using cat amplified from UT02, *inuABCDEF* was introduced into *B. subtilis* 168 and cultured on selection medium: minimal medium containing chloramphenicol (a), inulin (b), or both (c), at 37℃ for 24 hours.


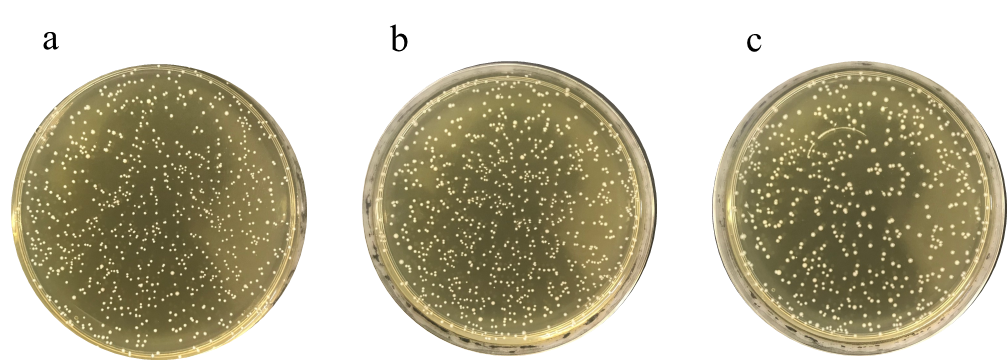


**Supplementary Fig. 5. Deletions of *sacA* (a), *levB* (b), and *sacC* (c) were respectively introduced into the UT02 strain, which was then selected on minimal medium containing erythromycin and lincomycin at 37℃ for 48 hours.** Established strains were designated UT03, UT04, and UT05, respectively.


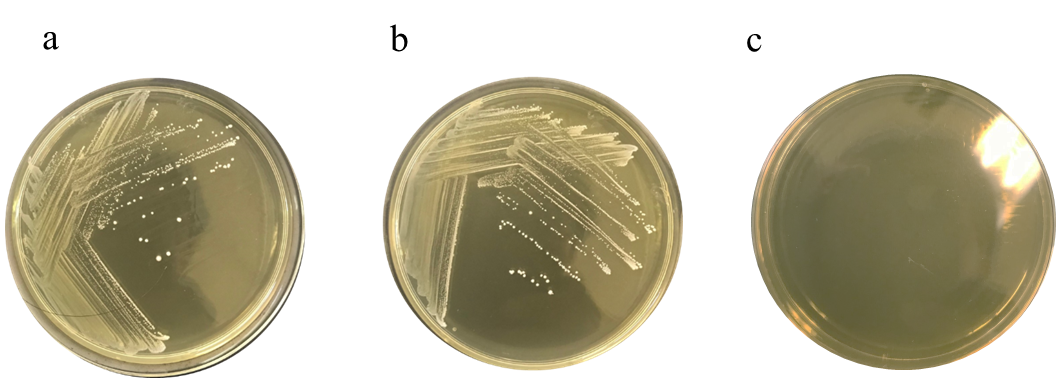


**Supplementary Fig. 6. The growth of UT03 (a), UT04 (b), and UT05 (c) strains on minimum media containing an inulin as the sole carbon source after 48 hours incubation at 37℃.**


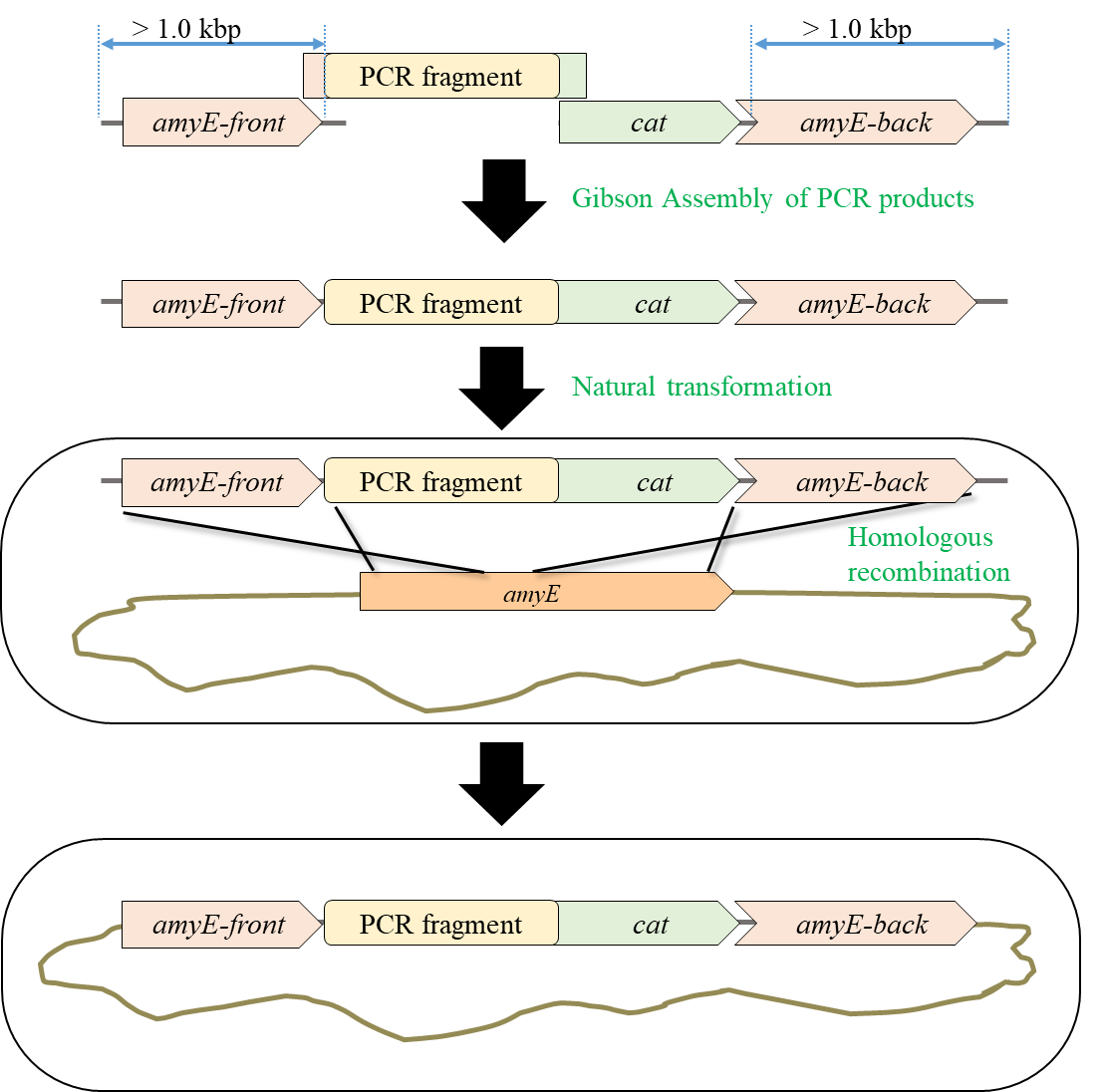


**Supplementary Fig. 7. Schematic representation of transformation to introduce candidate genes into *amyE* locus of *Bacillus subtilis* via double-crossover events.** Upstream and downstream of the *amyE* (*amyE-front* and *amyE-back*, respectively), chloramphenicol resistance gene (*cat*) from *B. subtilis* YK05 and Ldb1381-Ldb1386 or Ldb0438-Ldb0448 were assembled using the Gibson Assembly. Then, the assembled PCR fragments were introduced into *B. subtilis* by natural transformation to obtain the mutant strains indicated.
