# Supplementary material for: Identification of genes encoding a novel ABC transporter in Lactobacillus delbrueckii for inulin polymers uptake
Source: Sci Rep. 2021 Aug 6;11:16007. doi: 10.1038/s41598-021-95356-1 (PMC8346543; doi:10.1038/s41598-021-95356-1)
Supplement: Supplementary file 2 — Supplementary Information 2. [file 41598_2021_95356_MOESM2_ESM.docx]

**Identification of genes encoding a novel ABC transporter in *Lactobacillus delbrueckii* for inulin polymers uptake**

Yuji Tsujikawa, Shu Ishikawa, Iwao Sakane, Ken-ichi Yoshida, and Ro Osawa

Supplementary Table

**Supplementary Table 1.** 22 candidate genes selected as described in the method and their transcription levels on inulin compared to cellobiose by RNA-seq.

| Locus tag | Product | Fold change ^(a)^ | |
| --- | --- | --- | --- |
|  |  | Inulin 0→6 h | Inulin/Cellobiose |
| Ldb0021 | ABC transporter ATP-binding protein | 2.48 | 2.25 |
| Ldb0035 | putrescine/spermidine ABC transporter ATP-binding protein | 2.48 | 3.14 |
| Ldb0154 | ABC transporter ATP-binding protein | 4.53 | 2.95 |
| Ldb0276 | peptide ABC transporter substrate-binding protein | 3.63 | 2.19 |
| Ldb0295 | purine permease | 3.12 | 2.58 |
| Ldb0330 | ABC transporter permease | 7.31 | 3.18 |
| Ldb0331 | metal ABC transporter ATP-binding protein | 9.13 | 2.75 |
| Ldb0442 | PTS sugar transporter subunit IIC | 3.39 | 2.23 |
| Ldb0476 | hypothetical protein | 2.51 | 3.51 |
| Ldb0973 | ABC transporter ATP-binding protein | 33.59 | 2.77 |
| Ldb0974 | ABC transporter ATP-binding protein | 32.9 | 2.77 |
| Ldb1221 | M23 family peptidase | 7.26 | 2.36 |
| Ldb1298 | amino acid ABC transporter ATP-binding protein | 4.69 | 2.73 |
| Ldb1299 | ABC transporter permease | 2.99 | 2.75 |
| Ldb1384 | ABC transporter permease | 2.71 | 2.51 |
| Ldb1385 | ABC transporter ATP-binding protein | 3.27 | 2.5 |
| Ldb1386 | ABC transporter ATP-binding protein | 3.39 | 2.22 |
| Ldb1574 | MFS transporter | 4.99 | 3.25 |
| Ldb1651 | hypothetical protein | 35.75 | 3.25 |
| Ldb1652 | hypothetical protein | 30.27 | 3.43 |
| Ldb1990 | hypothetical protein | 5.31 | 2.11 |
| Ldb1993 | hypothetical protein | 7.31 | 2.57 |

(a) Inulin 0→6 h; the range of the gene expression levels on inulin from 0 hour to 6 hours, Inulin/Cellobiose; Inulin/Cellobiose; the range of the gene expression level on inulin compared to cellobiose.

**Supplementary Table 2.** List of primers used in validation of the expression data obtained from the RNA-seq experiment.

| Locus tag | Sequence |
| --- | --- |
| Ldb0394 | F:5'-AATTCGAAGACGCCATTGTC-3' |
|  | R:5'-TCGTGGTATGAACCGTCGTA-3' |
| Ldb0021 | F:5'-TTGTGGGAGTCCCTTTTCTG-3' |
|  | R:5'-TGAACTTGCAAATGCTGGAG-3' |
| Ldb0035 | F:5'-TCCTGCAAGACGTCTCCTTT-3' |
|  | R:5'-TGGACCAGATCCAGGCTATC-3' |
| Ldb0154 | F:5'-GGGATTAAAGGGAGCTTTGC-3' |
|  | R:5'-AATCCCTGTCAGCAGTTTGG-3' |
| Ldb0276 | F:5'-TACCGTTTGGGCAAGAACTC-3' |
|  | R:5'-ATCCCGGAGAAGAGGTAGGA-3' |
| Ldb0295 | F:5'-CGGTGGTCTCTTCAACACCT-3' |
|  | R:5'-AAGTCGACCTTGAGCAGCAT-3' |
| Ldb0330 | F:5'-CGTTGGGAATCGTTCTTAGC-3' |
|  | R:5'-TTCTGAGCGCCTTTAATTGG-3' |
| Ldb0331 | F:5'-CCAGCAGCAACGAGTACAAA-3' |
|  | R:5'-GCGTAGCTTTCCAGCAGAGT-3' |
| Ldb0442 | F:5'-TCACCCTAGCCATCCTCTTG-3' |
|  | R:5'-TGACGATCATGCCGATAAAA-3' |
| Ldb0476 | F:5'-GAAGACGTCTCACCCCTGAC-3' |
|  | R:5'-GAAGTGGGCTCGGTATATGG-3' |
| Ldb0973 | F:5'-ACTCTTTCTCCCGGCTTGAT-3' |
|  | R:5'-TGGGATCCTGAAAGGCATAG-3' |
| Ldb0974 | F:5'-TTCAGCTGGCTGGAAAAACT-3' |
|  | R:5'-TCCTTTAGTTCACGGGCATT-3' |
| Ldb1221 | F:5'-GCTGGTACATCTGGGTCGTT-3' |
|  | R:5'-ATCACCTTCATCGGGTTCAG-3' |
| Ldb1298 | F:5'-GGCCCATGAACTTTTAGCAA-3' |
|  | R:5'-ATTTCCGGGTCTAAGGCACT-3' |
| Ldb1299 | F:5'-TCAACAACTGCCAAAAGCAG-3' |
|  | R:5'-CGAAGATGACACCCAGGACT-3' |
| Ldb1384 | F:5'-TTTCTGGCTTTCGCTTTTGT-3' |
|  | R:5'-AAAACTCCCCAGAAGCCACT-3' |
| Ldb1385 | F:5'-CGAGTGCTTTGGACTTGTCA-3' |
|  | R:5'-AAATGTGCTCCGGATTCTTG-3' |
| Ldb1386 | F:5'-ATGACATGTACCCGCATGAA-3' |
|  | R:5'-ACATCACGGCAACTTCATCA-3' |
| Ldb1574 | F:5'-GTGACGGTTTTAGCGGACAT-3' |
|  | R:5'-GGTAAAAGGCAACCAGCAAA-3' |
| Ldb1651 | F:5'-CTGATTTCTCCGCCTTTTTG-3' |
|  | R:5'-CGGCAAAGTAGGGATCAAAG-3' |
| Ldb1652 | F:5'-GGCAGTCCTGACTTGCCTAC-3' |
|  | R:5'-AAACCTGGAGACCGGAAAAG-3' |
| Ldb1990 | F:5'-CTGGACGCTACTTGTTGCTG-3' |
|  | R:5'-CCATTTTCAAAGCGAACCAC-3' |
| Ldb1993 | F:5'-AATCGTATCGCTCGAGTTGC-3' |
|  | R:5'-TGGCTCAAGAACTTGTCTACG-3' |

**Supplementary Table 3.** List of primers used for introduction of Ldb1381-Ldb1386 or Ldb0438-Ldb0448 regions into *amyE* region in *Bacillus subtilis* 168 by double crossing over event.

| primer | Sequence | Template |
| --- | --- | --- |
| cat-amyEF-f1 | AATTCTCCAGTCTTCACATCGGTTTGAAAGGAGGAAGCGG | *Bacillus subtilis*168 |
| cat-amyEF-r | GCGACTCTACCCATGTCACTAGCTTGTCCGCAGAC | *Bacillus subtilis*168 |
| cat-amyEB-f | CCGGGAATTCTCATGTTTGACAGCTTATCATCGGC | *Bacillus subtilis* YK05 |
| cat-amyEB-r1 | GGGGAAGAGAACCGCTTAAGCCCGAGTCATTATATAAACC | *Bacillus subtilis* YK05 |
| Ldb1380F | GTCTGCGGACAAGCTAGTGACATGGGTAGAGTCGC AATATCGTCCGCACTGAAGG | *Lactobacillus delbrueckii* JCM 1002^T^ |
| Ldb1387R | GCCGATGATAAGCTGTCAAACATGAGAATTCCCGG GCAGCGTCTTCATCAGGAAT | *Lactobacillus delbrueckii* JCM 1002^T^ |
| Ldb0437F | GTCTGCGGACAAGCTAGTGACATGGGTAGAGTCGC ACGGCCCTGTCTTACAGCTA | *Lactobacillus delbrueckii* JCM 1002^T^ |
| Ldb0449R | GCCGATGATAAGCTGTCAAACATGAGAATTCCCGG GTATGAAGCCCGCGTGTATT | *Lactobacillus delbrueckii* JCM 1002^T^ |
